# Pulmonary microbiota signatures adjacent to adenocarcinoma, squamous cell carcinoma and benign lesion

**DOI:** 10.3389/fonc.2023.1163359

**Published:** 2023-06-09

**Authors:** Jinyou Li, Gang Wu, Ju Yang, Jiai Yan, Dan Li, Qinyue Wang, Yanping Xia, Jie Zhu, Baoliang Guo, Fengyue Cheng, Jing Sun, Hong Cao, Feng Zhang

**Affiliations:** ^1^ Department of Thoracic Surgery, Affiliated Hospital of Jiangnan University, Wuxi, Jiangsu, China; ^2^ Department of Thoracic Surgery, First Affiliated Hospital of Soochow University, Suzhou, Jiangsu, China; ^3^ Wuxi School of Medicine, Jiangnan University, Wuxi, Jiangsu, China; ^4^ Department of Nutrition, Affiliated Hospital of Jiangnan University, Wuxi, Jiangsu, China; ^5^ Functional Food Clinical Evaluation Center, Affiliated Hospital of Jiangnan University, Wuxi, Jiangsu, China; ^6^ Lifestyle-Medicine Strategy to Improve Outcome for Cancer patients (LIOC) Group, Chinese Society of Nutritional Oncology, Beijing, China; ^7^ Department of Infection Control, Affiliated Hospital of Jiangnan University, Wuxi, Jiangsu, China; ^8^ School of Bioengineering, Jiangnan University, Wuxi, Jiangsu, China; ^9^ School of Environment and Civil Engineering, Jiangnan University, Wuxi, Jiangsu, China; ^10^ Chinese Society of Nutritional Oncology, Beijing, China

**Keywords:** adenocarcinoma, lung cancer, pulmonary microbiota, ralstonia, squamous cell carcinoma

## Abstract

**Introduction:**

The occurrence and progression of lung cancer are influenced by pulmonary microbiota, yet the relationship between changes in the pulmonary microbiota and lung cancer remains unclear.

**Methods:**

To investigate the correlation between pulmonary microbiota and the signature of lung lesions, we analyzed the microbial composition at sites adjacent to the stage 1 adenocarcinoma, squamous carcinoma and benign lesion tissues in 49 patients by using 16S ribosomal RNA gene sequencing. We then conducted Linear discriminant analysis, receiver operating characteristic (ROC) curve analysis and PICRUSt prediction based on 16S sequencing results.

**Results:**

Overall, the microbiota composition at sites close to lung lesions showed significant differences between different lesion types. Based on the results of LEfSe analysis, *Ralstonia, Acinetobacter* and *Microbacterium* are the dominant genera of lung adenocarcinoma (LUAD), lung squamous carcinoma (LUSC) and benign lesions (BENL), respectively. Furthermore, we determined the diagnostic value of the abundance ratio of *Ralstonia* to *Acinetobacter* in adenocarcinoma patients through ROC curve analysis. The PICRUSt analysis revealed 15 remarkably different metabolic pathways in these lesion types. In LUAD patients, the increase of the pathway associated with xenobiotic biodegradation may be due to the continuous proliferation of microbe with degradation ability of xenobiotics, which implied that LUAD patients are often exposed to harmful environment.

**Discussion:**

The abundance of *Ralstonia* was related to the development of lung cancer. By measuring the abundance of microbiota in diseased tissues, we can distinguish between different types of lesions. The differences in pulmonary microbiota between lesion types are significant in understanding the occurrence and development of lung lesions.

## Introduction

Lung cancer has become a malignant tumor with the highest morbidity and mortality (1). In recent decades, the incidence of lung cancer has gradually increased, and the epidemic spectrum has changed. Currently, the incidence rate of adenocarcinoma has rapidly surpassed that of squamous cell carcinoma and other pathological types of lung cancer. The reason for the rapid increase in the incidence rate of lung adenocarcinoma is not clear; smoking remains the primary risk factor for lung cancer (2), while other risk factors include family history of lung cancer, estrogen, lifestyle, exposure to radioactive substances, and chronic inflammation of the lung caused by microorganisms (3–5). Recent studies have found that the steady state of the respiratory tract flora is essential for good health (6). Lee et al. (7) compared the microbial composition of bronchoalveolar fluid between patients with lung cancer and those with benign lesions, and the two genera (*Veillonella and Megasphaera*) were found to be relatively more abundant in patients with lung cancer. Hosgood et al. (8) observed that the diversity of respiratory tract microbial composition in non-smoking female lung cancer patients was significantly higher than that in the control group, and lung cancer cases had an enrichment of respiratory tract microbiota compared to controls, especially *Streptococcus*. Yu et al. reported that the microbial composition in squamous cell carcinoma tumor samples was different from that in adenocarcinoma (9). A recent study compared lung microbial composition between patients with lung cancer and healthy controls and reported that a unique lung microbiome is associated with tumor tissue. In squamous cell carcinoma samples with TP53 mutations, a higher abundance of *Acidovorax* was discovered. In addition, *Klebsiella, Comamonas, Acidovorax, Polarmonas*, and *Rhodoferax* genera were more frequently found in small-cell carcinomas, but they were not found in adenocarcinoma (10). However, previous studies investigating the relationship between lung cancer and respiratory tract microorganisms often relied on sample collection from sputum or bronchial washing fluid (BWF), which may be susceptible to contamination with microorganisms from the upper respiratory tract. Furthermore, as the bronchoscope often fails to reach the lesion site, samples may not accurately reflect the composition of microorganisms near the tumor. To better investigate the composition of microorganisms near the lung lesion, we studied the pulmonary microbiome of different pathologic types.

## Methods

### Participants and general information

A total of 49 inpatients who underwent pulmonary lesion resection in the Affiliated Hospital of Jiangnan University from June 2020 to June 2021 were selected, and the types of patients were benign lesions or stage 1 cancer. All patients provided informed consent and agreed to provide their resected lung tissue for analysis. This study was approved by the Ethics Committee of the Affiliated Hospital of Jiangnan University (Ethics No. 201889) and registered on the Chinese Clinical Trial Register as ChiCTR2000031192. Patients were excluded if they had received antibiotics within one month before the surgery, suffered from a pulmonary infection within one month, or suffered from small cell lung cancer, pulmonary tuberculosis, pulmonary cryptococcosis, or received long-term immunosuppressive drug treatment (11).

### Sample collection and processing

Immediately after isolating the lung tissue, para-cancerous tissue or adjacent tissue within 2 cm of the benign lesions, measuring about 1 ∗ 1 cm in size, was collected and placed in dry ice, then transferred to the −80°C deep-frozen refrigerator. After thawing, the lung tissue was cut open and rinsed with sterile normal saline to collect the flushing fluid. According to histological classification, all tissues were divided into three groups: benign lesion (BENL) group, squamous cell carcinoma (LUSC) group, and adenocarcinoma (LUAD) group. Benign lesions include pulmonary bullae and fibromas.

### DNA extraction and genomic sequencing

Total DNA was extracted from samples using the E.Z.N.A. ^®^ Soil DNA Kit (Omega Bio-tek, Norcross, GA, U.S.). The quality of the genomic DNA was detected by 1% agarose gel electrophoresis, and the concentration and purity of the genome were determined by NanoDrop2000 (Thermo Fisher Scientific). The V3–V4 region of the 16S rRNA gene was amplified by PCR using primers 338F (5’-ACTCCTACGGGAGGCAGCAG-3’) and 806R (5’-GGACTACHVGGGTWTCTAAT-3’) (12). PCR amplification was performed in triplicate for each sample using a reaction mixture (20 μl), which contains 4 μl of 5×TransStart FastPfu buffer, 10 ng of template DNA, 0.5 μM of each primer, 2 μl of 2.5 mM dNTPs, and 0.4 μl of TransStart FastPfu DNA polymerase. The PCR procedure included an initial denaturation step at 95°C for 2 min, subsequent 29 cycles of temperature gradient at 95°C for 30 s, 55°C for 30 s, and 72°C for 30 s, an extension step at 72°C for 10 min, and finally a preservation step at 4°C in a GeneAmp 9700 thermocycler (ABI, USA). The triplicate amplicons were pooled together and recovered with a 2% (w/v) agarose gel, purified using an AxyPrep DNA Gel Extraction Kit (Axygen Biosciences, Union City, CA, USA), electrophoresed on a 2% (w/v) agarose gel, and the purified amplicon was quantified using a Quantus™ Fluorometer (Promega, USA). Under the same system (the same experimental steps and reagents), each barcore was set up as a blank negative control sample for the PCR process, with a total of 24 CK samples for follow-up experiments.

A composite sequencing library was constructed from the purified amplicon using the NEXTFLEX Rapid DNA-Seq Kit: (1) connector link; (2) magnetic bead screening was used to remove the self-connected fragments of the joint; (3) the library template was enriched by PCR amplification; and (4) the PCR products were recovered by magnetic beads to obtain the final library. The blank control group underwent the same process of risk database construction and sequencing. The libraries were sequenced on an Illumina Miseq sequencing platform (Illumina, San Diego, USA). The original data was uploaded to the NCBI SRA database (serial number: SRP * * *).

### Bioinformatics analysis

The quality of original paired-end sequencing sequences was controlled using fastp (13) (https://github.com/OpenGene/fastp, version 0.19.6) and combined using FLASH (14) (http://www.cbcb.umd.edu/software/flash, version 1.2.11). Sequences were then dereplicated and clustered *de novo* into operational taxonomic units (OTUs) at 97% similarity using UPARSE (15) (http://drive5.com/uparse/, version 7.1). Taxonomic assignment was performed by the RDP classifier (16) (http://rdp.cme.msu.edu/, version 2.11) using the default confidence level of 0.7 against the Silva 16S rRNA database (v138). Then statistical analyses were conducted using the R package decontam (https://github.com/benjjneb/decontam), and the prevalence-based strategy was selected to eliminate the possible pollution annotated in the environmental samples by using the high-frequency species of the negative control samples (17). Finally, the number of sequences in all samples was flattened, and then the community composition of each sample was counted at different species classification levels to minimize the impact of sequencing depth on subsequent alpha and beta diversity data analysis. PICRUSt2 (18) (version 2.2.0) was used for 16S function prediction analysis.

## Results

### Characteristics of the study participants

A total of 49 patients were divided into three groups based on the histological classification of the lesions: 11 participants in the BENL group, nine participants in the LUSC group, and 29 participants in the LUAD group. The baseline data (gender, age, and smoking index) of participants in all three groups were statistically analyzed. The smoking index was calculated by multiplying the number of cigarettes per day by the number of years of smoking. There was no statistically significant difference in gender distribution, age, or smoking index among the three groups (*P >*0.05) (**Table 1**).

**Table 1:** Baseline characteristics.

	BENL (n = 11)	LUSC (n = 9)	LUAD (n = 29)	*P*
male/female	5/6	3/6	9/20	*P* = 0.908
Age	59.5 ± 6.68	59.89 ± 7.72	61.24 ± 6.83	*P* = 0.695
Smoking index	248.75 ± 346.02	265 ± 315.71	261.21 ± 434.12	*P* = 0.993

### α-diversity

**Figure 1:**
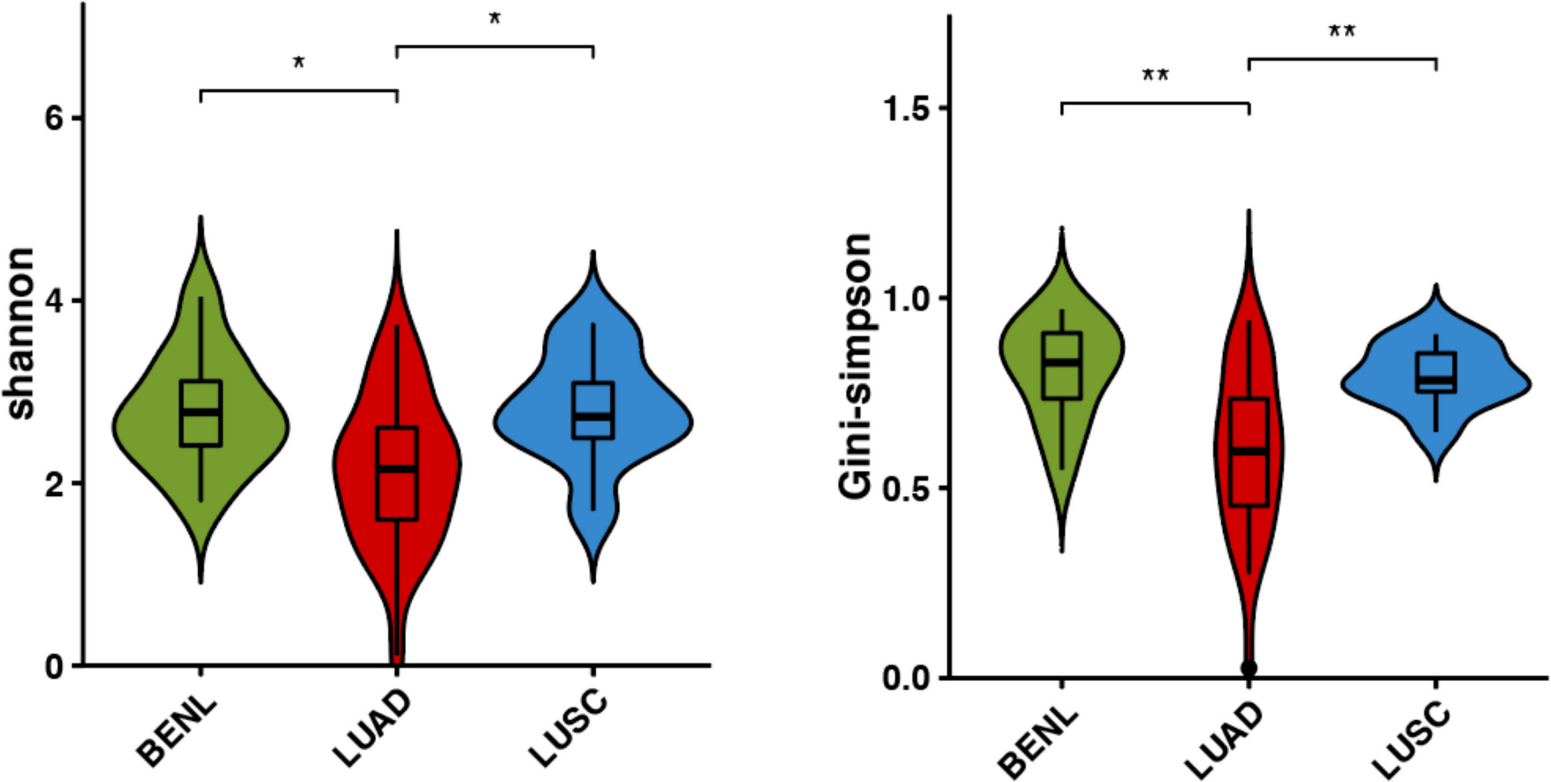
α-diversity in bacterial communities in three classifications of lung lesions. Shannon index and Simpson index were indexes to evaluate α-diversity, and there were differences in the LUAD group, BENL group, and LUSC group. *P<0.05, **P<0.01.

According to the Shannon index and the Gini-Simpson index, the α-diversity of the pulmonary microbiota in three lung lesions was analyzed. Statistical analysis revealed a significant difference in α-diversity between the BENL group and the LUAD group, as well as between the LUSC group and the LUAD group, while there was no statistical difference between the BENL group and the LUSC group. Overall, the pulmonary microbiota in the LUAD group exhibited the lowest diversity (**Figure 1**).

### β-diversity

**Figure 2:**
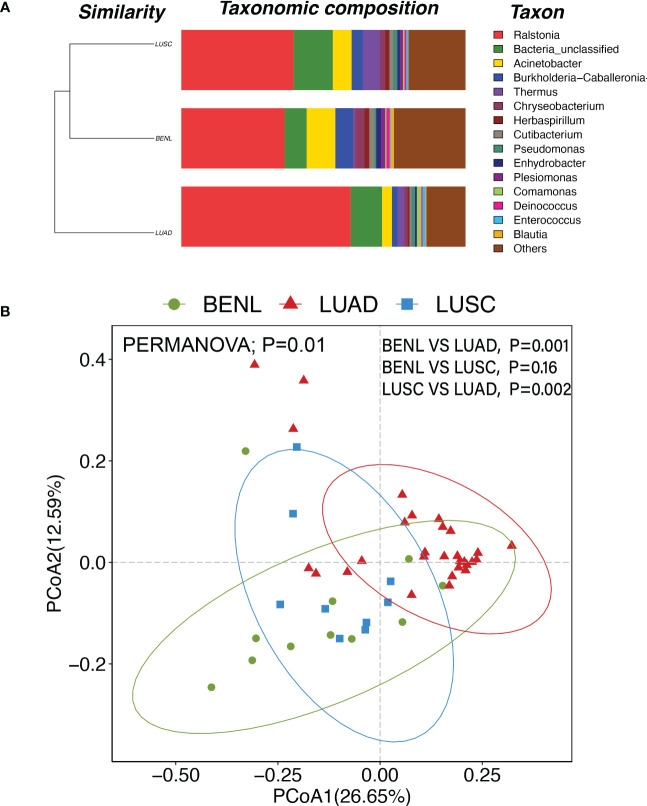
**(A)** At the genus level, the LUSC group and the BENL group had higher similarity and a closer cluster analysis distance, while the LUAD group was far apart and had poor similarity compared with the LUSC and BENL groups. **(B)** PCoA diagram and PERMANOVA analysis showed that there were significant differences in pulmonary microbial structures between LUAD and BENL groups (P = 0.001), LUAD and LUSC groups (P = 0.002), but no significant differences between LUSC and BENL groups (P = 0.16).

Clusters of pulmonary bacterial genera were identified in hierarchical clustering of BENL, LUSC, and LUAD samples, indicating that pulmonary microbiota in the LUSC group were similar to those in the BENL group, while the LUAD group had an obvious different microbial structure compared with the BENL and LUSC groups (**Figure 2A**). The β-diversity of pulmonary microbiota based on Principal Coordinates Analysis (PCoA) showed similar differences with hierarchical clustering resulting in structures between three groups (**Figure 2B**). PERMANOVA analysis suggested that pulmonary microbial structure in the LUAD group showed a significant difference from that in the BENL and LUSC groups (LUAD vs. BENL, *P* = 0.001; LUAD vs. LUSC, *P* = 0.002), while no difference in microbiota structure was observed between the BENL and LUSC groups (*P* = 0.16).

### The representative taxa in each group

**Figure 3:**
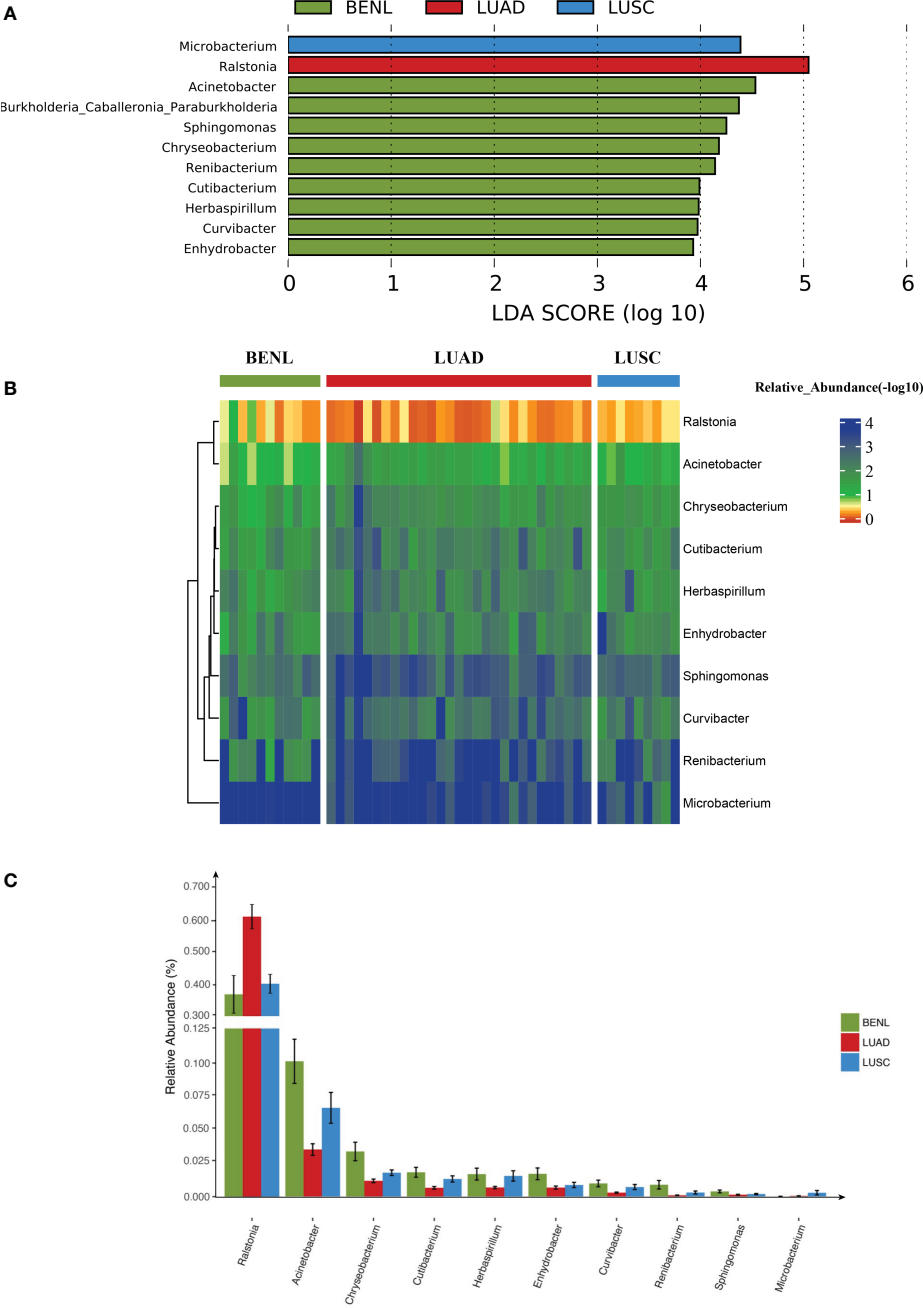
Lung microbiota in lung cancer and relevance. **(A)** The abundance of *Ralstonia* in the LUAD group was significantly higher than that of BENL and the LUSC group by statistical analysis of the top three genera. **(B)** The abundance of *Microbacterium* in the LUSC group was significantly higher than that in the LUAD and BENL groups. The relative abundance of the displayed microbes was presented in a heat map (−log 10). Different colors corresponded to different abundances; smaller numbers corresponded to higher abundances. **(C)** The abundance of *Acinetobacter in the* BENL group was significantly higher than that in the LUSC and LUAD groups.

The linear discriminant analysis (LDA) effect size (LEfSe) method was used to screen out key OTUs that could discriminate among three groups. *Ralstonia*, *Microbacterium*, and *Acinetobacter* were representative taxa in the LUAD, LUSC, and BENL groups, respectively (**Figure 3A**). The abundance of *Ralstonia* in the LUAD group was significantly higher than that in the BENL and LUSC groups. The abundance of *Microbacterium* in the LUSC group was higher than that in the LUAD and BENL groups. The abundance of *Acinetobacter in the* BENL group was significantly higher than that in the LUSC and LUAD groups (**Figures 3B, C**).

### ROC curve analysis

**Figure 4:**
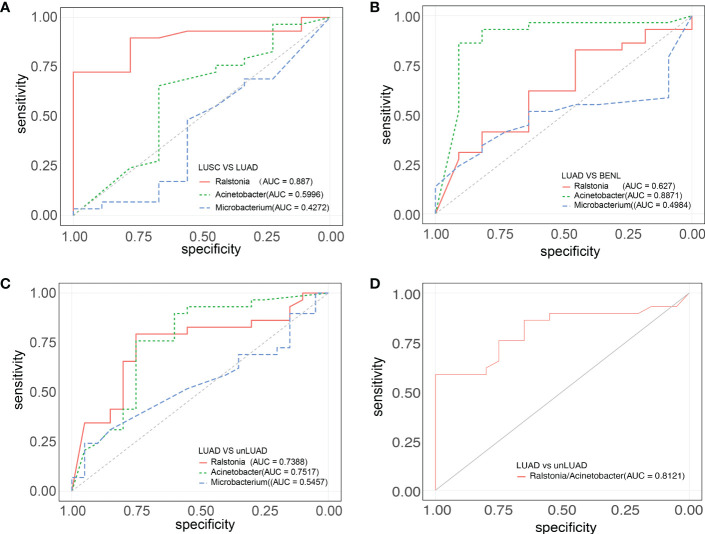
ROC curves of the three groups of highly abundant strains. **(A)** *Ralstonia* (AUC = 0.887) had good specificity and sensitivity to distinguish LUAD and LUSC. **(B)** *Acinetobacter* (AUC = 0.8871) has good specificity and sensitivity to distinguish between LUAD and BENL. **(C)** The prediction effects of *Ralstonia* (AUC = 0.7388) and *Acinetobacter* (AUC = 0.7517) were good and had certain diagnostic value. **(D)** The abundance ratio of *Ralstonia* to *Acinetobacter* (AUC = 0.8121) can well predict LUAD and unLUAD.

The receiver operating characteristic (ROC) curve (AUC) between lesion specimens was used to evaluate the prediction accuracy of three genera (*Ralstonia*, *Acinetobacter*, and *Microbacterium*), which were screened out by LEfSe. *Ralstonia* (AUC = 0.887) had good specificity and sensitivity in distinguishing the LUAD and LUSC groups. Between the LUAD and BENL groups, *Acinetobacter* (AUC = 0.8871) was the best predicted genus. *Acinetobacter* (AUC = 0.7517) was the best genus for discriminating LUAD (vs. unLUAD), followed by *Ralstonia* (AUC = 0.7388). Since the abundance of *Ralstonia* was higher while that of *Acinetobacter* was lower in the LUAD group compared with that in the LUSC and BENL groups, we attempted to use the abundance ratio of *Ralstonia* to *Acinetobacter* to predict LUAD more efficiently. As expected, this ratio (AUC = 0.8121) can well predict LUAD (**Figure 4**).

### PICRUSt prediction based on 16S sequencing results

**Figure 5:**
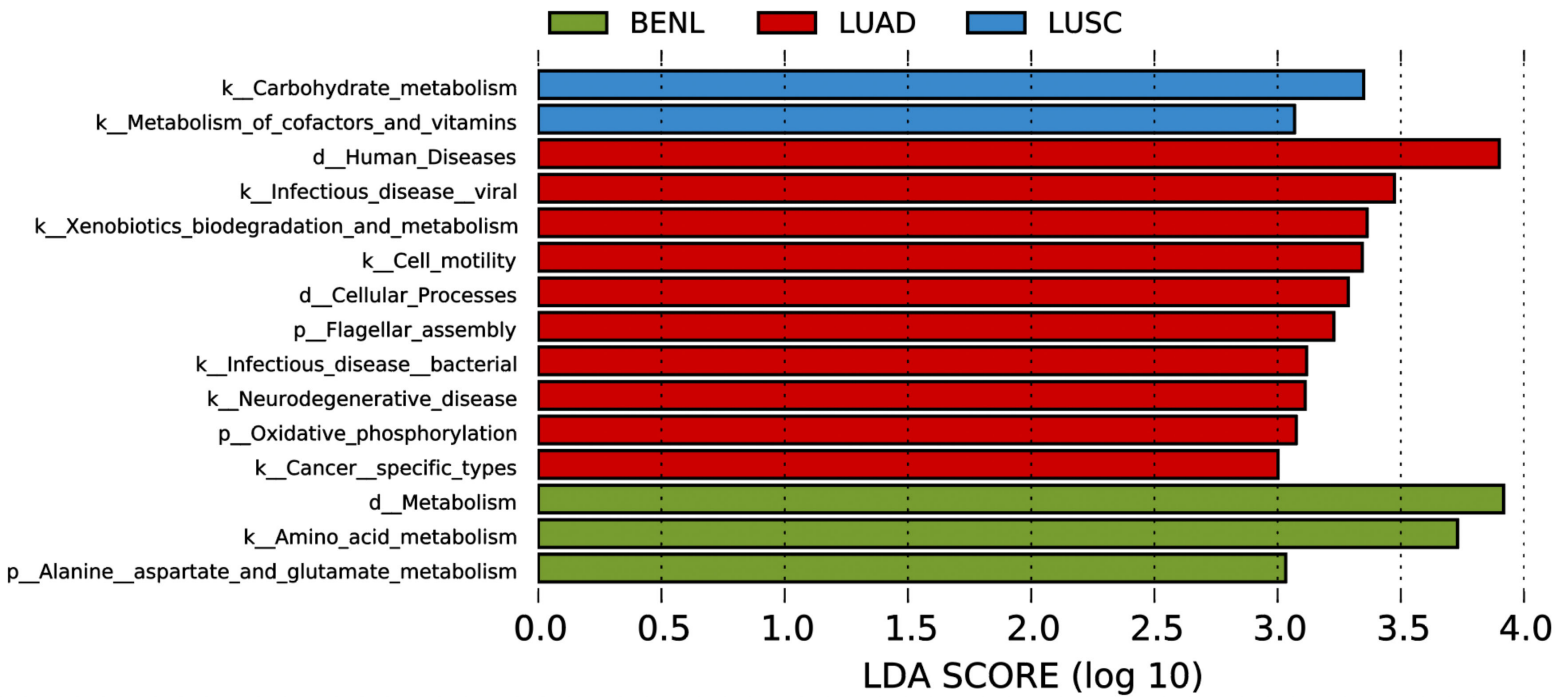
PICRUST-based lung microbiota study in patients with LUSC, LUAD, and BENL. Results are colored by the lesion’s category and sorted in decreasing order of degree of increase within each category. Note that the LDA score (log 10) was >3.0, and the region 3.0–4.0 displayed pathways with more increase.

In the functional annotation analysis based on 16S rRNA gene data by PICRUSt, we found an enrichment in the representation of pathways associated with carbohydrate, cofactors, and vitamin metabolism in the LUSC group; pathways associated with alanine, aspartate, and glutamate metabolism in the BENL group; and pathways associated with flagellar assembly, xenobiotic biodegradation and metabolism, and oxidative phosphorylation in the LUAD group (**Figure 5**).

## Discussion

Under normal circumstances, the symbiotic microbial community is beneficial to human health. However, disordered microbial communities result in changes in the local microenvironment and induce the body’s immune response (19), leading to various diseases, the most serious of which are malignant tumors. Previous studies have shown that a variety of malignancies are directly caused by potential gastrointestinal opportunistic microorganisms, such as *Helicobacter pylori*, which is known to promote the occurrence and progression of gastric cancer (20, 21), and the amount of *Fusobacterium nucleatum* in colorectal carcinoma has been associated with shorter patient survival (22). Thus, the microbiota plays an important role in the development of colon cancer.

The change in lung microorganisms also has a certain correlation with the occurrence and development of lung cancer. Germ-free rats were found to be less likely to develop lung cancer than infected rats (23). *Capnocytophaga*, *Selenomonas*, *Veillonella*, and *Neisseria* were found to be significantly altered in the saliva of patients with squamous cell carcinoma and adenocarcinoma (24). However, it is important to note that many of these studies have used samples collected from bronchoalveolar lavage fluid, sputum, or bronchial washing fluid, which may have different microbial compositions compared to samples taken closer to the tumor site. Therefore, collecting samples closer to the tumor site may provide a more accurate microbial composition analysis (25). Bronchoalveolar lavage fluid and bronchial washing fluid can seldom reach the alveoli of peripheral lung cancer, and the specimen would be contaminated by bacteria in the proximal bronchus. In lung cancer tissue, the abundance of bacteria is low, and the tumor tissues we collected are mostly stage 1 cancer with a small volume, which is insufficient for microbiome analysis after pathological examination. Microbiota may be mainly collected from the alveoli adjacent to lesion in our study.

It has been suggested that a lower α-diversity of respiratory microbiota was observed in patients with malignant tumors (26). In this study, the pulmonary microbiota in the LUAD group had the lowest diversity. According to β-diversity and hierarchical cluster analysis, pulmonary microbial structure in the LUAD group showed a significant difference from that in the BENL and LUSC groups, while no significant difference in microbial structure was observed between the BENL and LUSC groups.

LEFSe analysis showed that the abundance of *Ralstonia* in the LUAD group was significantly higher than in the BENL group and the LUSC group. *Microbacterium* and *Acinetobacter* were the dominant bacteria in the LUSC group and the BENL group, respectively. These findings suggest that the composition of the microbiota close to tumor tissues has changed. It has been reported that *Ralstonia* and *Helicobacter* were the dominant bacteria before gastric cancer surgery, while *Streptococcus* and *Prevotella* were the dominant bacteria after the operation (27). It has also been found that *Ralstonia* can be used as a prediction model for biliary tract cancer (28). We discovered that the abundance ratio of *Ralstonia* to *Acinetobacter* can better predict non-LUAD and LUAD, while *Ralstonia* could be used to distinguish LUAD from LUAC based on the ROC curve. Therefore, we speculate that *Ralstonia* may play an important role in the occurrence and development of LUAD. By predicting the metabolic pathways of microbiota, we found that compared with the metabolic pathways associated with LUSC and BENL, some specific pathways associated with lung cancer were discovered to increase in LUAD, such as those associated with bacterial infection, xenobiotic biodegradation, and oxidative phosphorylation.

The increase in the pathway associated with bacterial infection has been observed in many cancer studies. Lung cancer has been linked to inflammatory diseases, and lung-specific carcinogenesis is partly associated with a persistent local inflammatory state (29, 30). Inflammation in the tumor microenvironment has many tumor-promoting effects (31). Neutrophils infiltrating mouse tumors can promote carcinogenesis by supporting tumor-related inflammation in mouse models of lung cancer (32). The increase in the pathway associated with xenobiotic biodegradation may be related to the patient’s exposure to more xenobiotics (33). Lee et al. reported that the abundance of *Ralstonia* increased after the use of cosmetics, indicating that *Ralstonia* in facial skin may have the capacity to metabolize cosmetic components (34). Some strains of *Ralstonia* can degrade aromatic hydrocarbons, trichloroethylene, and quinoline in contaminated environments, and the toxicity of the degraded products is weaker compared with the original substances (35). The increase in the oxidative phosphorylation pathway in the LUAD group is also consistent with the pathological process of lung cancer. Studies have concluded that the increase in oxidative phosphorylation is related to the shorter survival time of lung adenocarcinoma patients (36). Lin et al. found that an actin-binding protein can promote lung cancer metastasis and colonization by enhancing mitochondrial oxidative phosphorylation (37). The application of oxidative phosphorylation inhibitors can slow down the metastasis and invasion of mouse malignant tumor cells (38). Deribe et al. found that oxidative phosphorylation (OXPHOS) was enhanced in smarca4 mutant tumors (39).

It has been proven that *Ralstonia*, an opportunistic pathogen, can accelerate the progression of cystic fibrosis when immunocompromised, making it a worse prognostic marker (40). *Ralstonia* is found in both water and soil and can infect a wide range of plants globally (41). For humans, *Ralstonia* is typically considered a low-pathogenic organism. The potential impact of *Ralstonia* on LUAD still requires further investigation to elucidate the reason for this bacterium being a dominant member near the lesion of LUAD and its role in degrading xenobiotics.

This study innovatively takes the alveoli tissue adjacent to the lesion as the sample, which can more accurately reflect the changes in the microbial environment adjacent to the tumor than bronchoalveolar lavage fluid. Further research is called upon to provide a new mechanism related to the occurrence and development of lung adenocarcinoma and provide new targets and methods for the prevention and treatment of LUAD. However, this study also has a few limitations: 1. The number of samples is modest: a larger sample size may lead to more accurate results. 2. Because most of the extractive genes in tissue flushing fluid are derived from the host, this study did not perform metagenomic sequencing analysis. However, we believe that the results of this study are still of great significance in understanding the role of pulmonary microbiota in the occurrence and development of lung cancer since these tumor tissues are from stage 1 cancer.

In conclusion, the microbial structure in patients with LUAD was significantly different from that in LUSC and BENL. *Ralstonia* might have an impact on the development of lung adenocarcinoma. We speculate that the increase in the pathway associated with xenobiotic biodegradation in patients with adenocarcinoma may be related to their exposure to more xenobiotics. Notably, by measuring the abundance of microbiota in pathological tissues, we can predict the types of lesions. As the histopathologic examination of the tissue is the conventional method, the abundance ratio of *Ralstonia* to *Acinetobacter* was used in the study of the significance of the change of microbiota in the occurrence and development of lung adenocarcinoma rather than in the prediction of the type of lesion.

## Data availability statement

The original contributions presented in the study are included in the article/supplementary material, further inquiries can be directed to the corresponding authors.

## Ethics statement

The studies involving human participants were reviewed and approved by Ethics Committee of Affiliated Hospital of Jiangnan University (Ethics No. 201889) and registered on the Chinese Clinical Trial Register as ChiCTR2000031192. The patients/participants provided their written informed consent to participate in this study.

## Author contributions

FZ, HC conceived the studies of pulmonary microbiota in lung cancer, designed experiments and wrote the manuscript; JL collected samples and analyzed the data; GW performed 16S rRNA gene sequencing analysis and wrote the manuscript; QW, JY, GW, BG, FC performed statistical analysis; DL, JAY, YX, JS, J.Z helped analyze the data; All authors reviewed, revised and approved the manuscript for submission.
